# Cross-platform and cross-interaction study of user personality based on images on Twitter and Flickr

**DOI:** 10.1371/journal.pone.0198660

**Published:** 2018-07-11

**Authors:** Zahra Riahi Samani, Sharath Chandra Guntuku, Mohsen Ebrahimi Moghaddam, Daniel Preoţiuc-Pietro, Lyle H. Ungar

**Affiliations:** 1 Faculty of Computer Science and Engineering, Shahid Beheshti University G.C, Tehran, Iran; 2 Positive Psychology Center, University of Pennsylvania, Philadelphia, PA, United States of America; 3 School of Medicine, University of Pennsylvania, Philadelphia, PA, United States of America; 4 Computer & Information Science, University of Pennsylvania, Philadelphia, PA, United States of America; University of Texas at San Antonio, UNITED STATES

## Abstract

Assessing the predictive value of different social media platforms is important to understand the variation in how users reveal themselves across multiple platforms. Most social media platforms allow users to interact in multiple ways: by posting content to the platform, liking others’ posts, or building a user profile. While prior studies offer insights into how language use differs across platforms, differences in image usage is less well understood. In this study, we analyzed variation in image content with user personality across three interaction types (posts, likes and profile images) and two platforms, using a unique data set of users who are active on both Twitter and Flickr. Usage patterns on these two social media platforms revealed different aspects of users’ personality. Cross-platform data fusion is thus shown to improve personality prediction performance.

## Introduction

According to a Pew Research study [1], 56% of US adults online use more than one social media platform. While some of these, such as LinkedIn have a specific use [2], other platforms such as Twitter are used in diverse ways by different groups of users [3]. Also, there are multiple ways in which users can interact with a social media platform—either by posting content to the platform, liking content that others have posted or maintaining up their user profile.

The volume and diversity of content that users produce and exchange on social media has led to the possibility of performing computational analysis and prediction of users’ personality based on their social media footprints [4]. While several studies focused on one social media platform and type of interaction, such as liked pages on Facebook [5], very few studies considered cross-platform data to analyze personality differences [6, 7]. Moreover, no study to date examined the different types of interactions (termed as ‘modalities’ in the rest of this text) performed on the same platform such as posted, liked and profile content.

With images gaining popularity in social media posts, personality traits can be inferred based on image-based content analysis. Images contain various concepts such as scenes, objects, colors or faces and these can be automatically captured using current computer vision algorithms. These representations can be used to analyze the relationship between users’ personality and image posting across different modalities and social media platforms.

Prior research [8] suggests that personality is strongly expressed on a platform which offers users sufficient self-expression and freedom of control. Social media platforms offer users the opportunity to have multiple types of interactions. These modalities reveal more complex and diverse patterns of behavior. Consequently, exploring different interactions that users have on social media platforms might provide a better understanding of users’ personality.

The aim of this paper is to quantify image sharing preferences and to build models that automatically predict users’ personality in a cross-/modal and cross-platform setting.

**Research Questions:** The research questions motivating this study are:

- *How are personality traits related to what images users post, like and set as profile picture?* We term these as different modalities of interaction with the platform.
- *How are personality traits expressed differently across platforms through images?* We use a set of users who have active accounts on both Flickr and Twitter.
- Can combining data from multiple platforms help improve performance of automatically predicting user personality?

Computational models that predict user traits based on their online footprints have several applications in targeted online marketing, increasing acceptance of HCI systems, personalized search and recommendations and exploring social science hypotheses based on large-scale social media data.

## Related work

With proliferation of mobile technologies and image sharing platforms, sharing pictures is the most commonly action (82% of the American users), followed by exchanging text messages (80% of the users) and accessing the Internet (56% of the users) [9]. In other words, “photos have become an important social content online [10, 11] that and can serve as a substitute for more direct forms of interaction like email [12].

This work contributes to recent social media trends that try to consider the content of their users’ interaction to predict personality of their users. Personality is a combination of all the attributes which includes differences in human behavior, thinking and feeling. Identifying personality of people has always been of great interest due to its importance. Personality traits have influence on many aspects of user behavior such as job performance [13], music preferences [14], psychological conditions [15–17], leadership ability [18], academic abilities and motivation [19], emotional responses to multimedia [20, 21], sales ability [13], perception of multimedia quality [22–24] and so on.

Recent research has examined the interplay between users’ personality traits—usually measured using the Big Five model [25, 26]—and their social media data [27]. Facebook *page likes* [5] and status updates [28] were used to accurately infer users’ personal information. Users choice to disclose particular sections of their social media profile was used to study their personality traits [29]. Images on social media are now increasingly being used due to their increased production and exchange in the recent years [4].

Most of previous research on images focused solely on profile images using facial features. Self-assessed personalities of 100 users were predicted using their Facebook profile images [30] with ∼65% accuracy using bag-of-visual-words features. Random portraits from the web [31] and existing face recognition data sets [32] were also used to model users’ personality. Recently, aesthetic features in addition to facial features were also used to study and predict personality on a ∼66,000 user data set [33] from Twitter.

Multiple platforms have recently been studied to infer users’ personality. For instance, an attempt to fuse cues from Instagram and Twitter reported a consistent decrease of the prediction errors for each personality trait [6]. Also in a cross-platform scenario involving Instagram and Twitter, [7] studied the differences in topics. However, no prior study examined the different types of user interactions such as posts, likes and profile images.

Table 1 shows a comparison of most recent studies in this area. They use variety of image features from different modalities of activity such as (profile, posted or liked) on different platforms to predict personality. In this paper we do a cross-platform and cross modality analysis to predict personality from social activities, comparing and contrasting the predictive value of each.

**Table 1:** Characteristics of recent work in Image-based personality analysis on social media.

Study	# Users	Assessment	Image Type	Social Platform	Image Features
Ferwerda et al. [34]	193	Perceived Personality	Posted Images	Instagram Photos	Content
Ferwerda et al. [29]	113	Self-assessed	posted images	Instagram Photos	Colors, #Faces, Filters
Liu et al. [33]	66,502 + 434	Self-assessed and estimated	Profile images	Twitter	Color, Facial
Nie et al. [35]	2238	Perceived	Portrait Images	Google	Facial, Social information
Guntuku et al. [36]	4132 + 161	Estimated and self-assessed	Posted, liked images and text	Twitter	Color, Bag of Imagga tags, VGG-Net trained on 1000 object categories
Guntuku et al. [37]	123	Self-assessed and perceived	Selfies (self-portraits)	Weibo	Color, Aesthetics, GIST, LBP, Bag-of-Visual-Words, Abstract sentiments, Fisher encodings of SIFT, SURF, HOG and MSER
Guntuku et al. [38, 39]	300	Self-assessed and perceived	Liked (‘Fave’) images	Flickr	Colors, semantic features, aesthetics
Wei et al. [40]	3,162	Self-assessed	Profile and posted images	Weibo	Colors, CNN, text
Nie et al. [41]	1000	Social Media Behavior	Portrait images	Micro Blogs	Concept and Emotion Detector, Active period, Level of attention (interests), and frequency of posts and forwards
Sang et al. [42]	300	Self-assessed	Liked (‘Fave’) images	Flickr	Aesthetics and Content feature
Segalin et al. [43]	300	Self-assessed	Liked (‘Fave’) images	Flickr	Convolutional Neural Network
Segalin et al. [44]	300	Self-assessed	Liked (‘Fave’) images	Flickr	Color, Composition, Texture, No of Faces
Segalin et al. [45]	11,736	Self-assessed	Profile images	Facebook	Aesthetics, BOVW, VGG-Net, IATO
Skowron et al. [6]	62	Self-assessed	Posted images, text users’ meta features	Twitter and Instagram	Pleasure-Arousal-Dominance, Color, Face, Body, Textual tags, followers and publicly available counts
Xiong et al. [46]	300	Self-assessed	Liked (‘Fave’) images	Flickr	Faces, Color, Composition, Texture, Gist
AlMoubayed et al. [32]	829	Perceived personality	Face Images	Face Recognition Database	Eigenfaces
Celli et al. [30]	112	Self-assessed	Profile Images	Facebook	Bag of Visual Words (BOVW)

## Materials and methods

**Fig 1:**
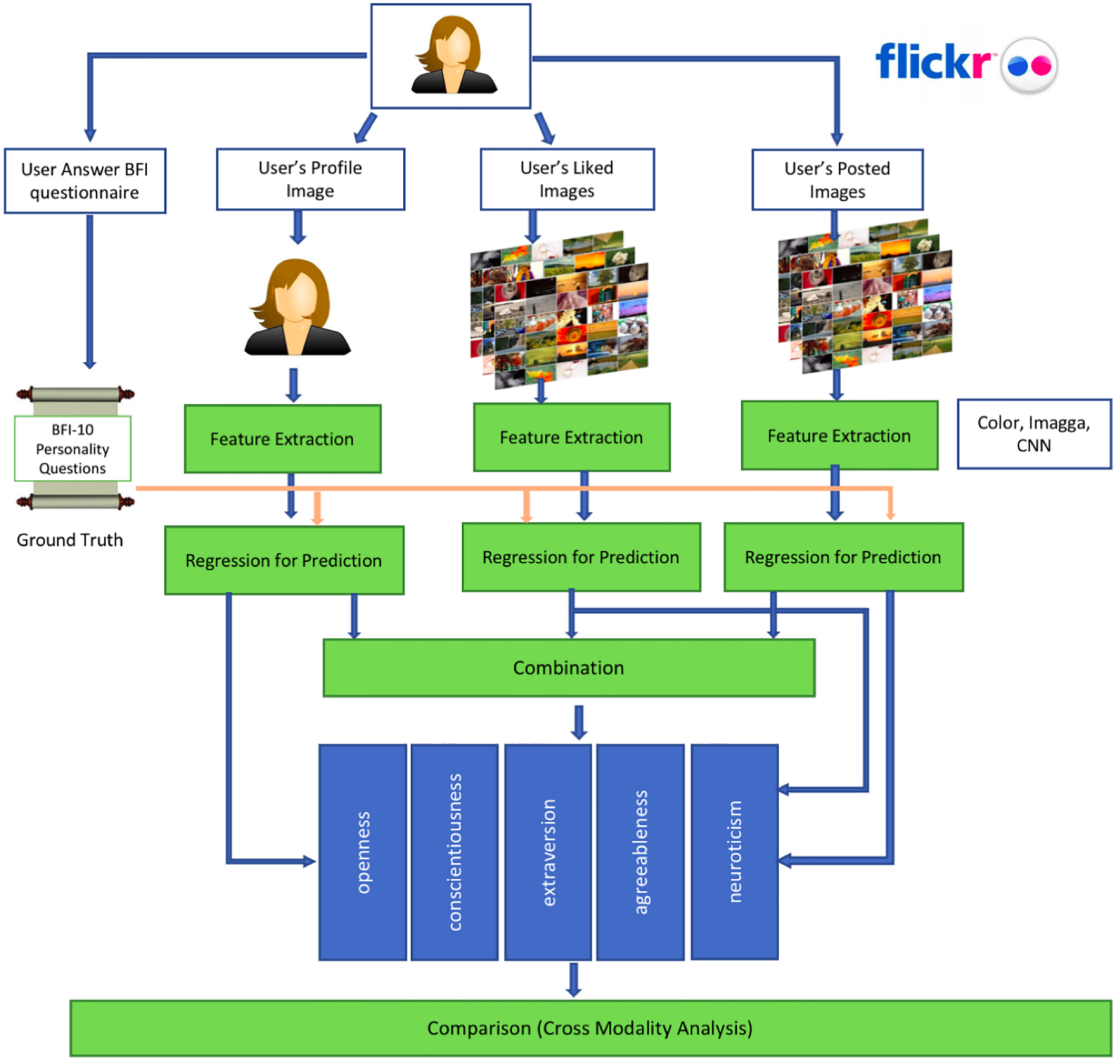
Overview of cross-modal analysis.

**Fig 2:**
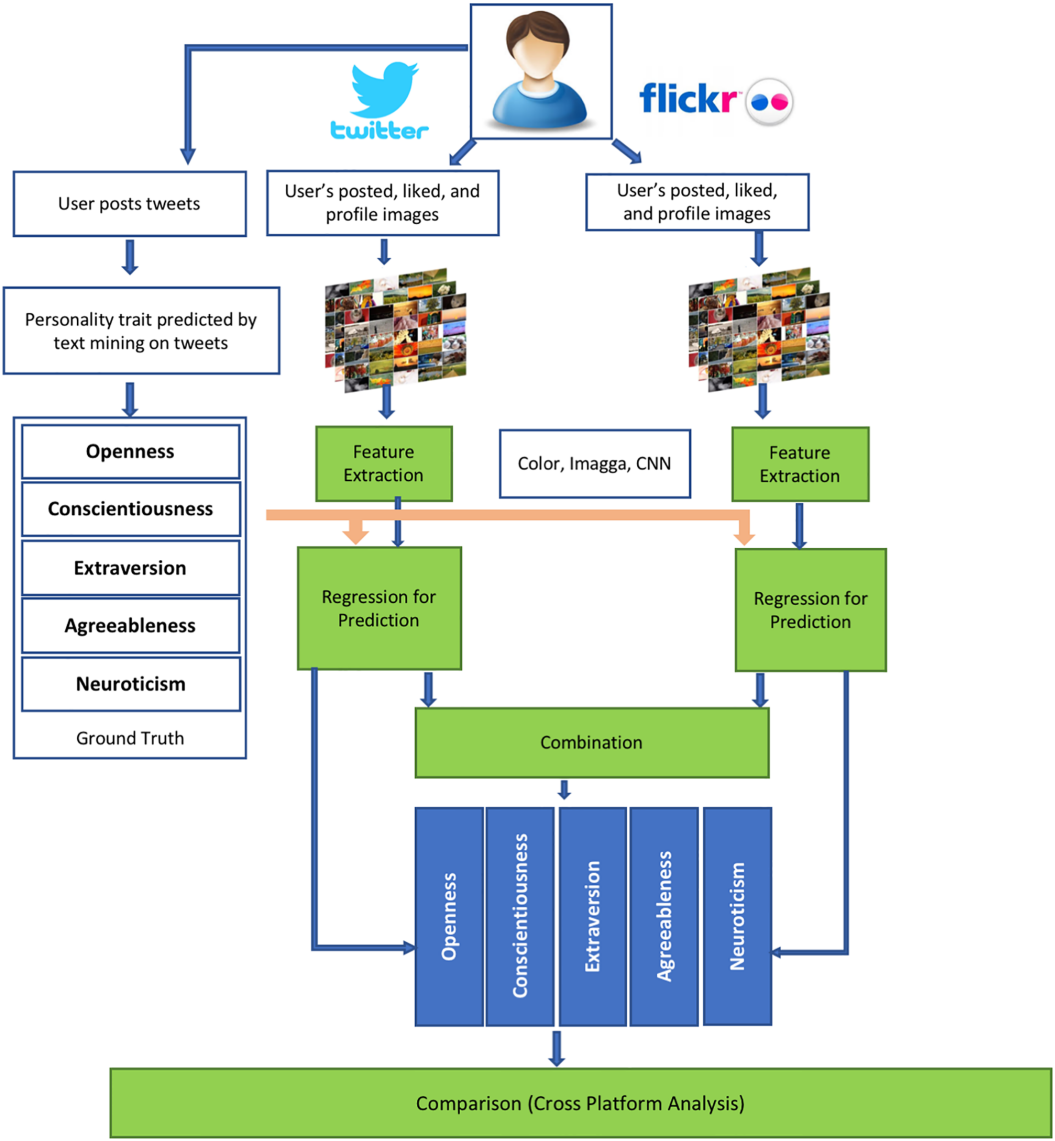
Overview of cross-platform analysis.

We use two data sets in our experiments. The first data set contains a set of Flickr users with their self-assessed personality traits. This data set is used to compare the predictive power of various image interactions of these users on Flickr. The second data set is built for this study and consists of users who have active accounts both on Twitter and Flickr. Personality traits for this group are estimated by analyzing their online text. Image interactions of these users on both platforms are used in cross-modal and cross-platform analysis. Figs 1 and 2 show the process of our cross-modal and cross-platform analysis. We also compare different features in predicting personality traits and perform experiments to uncover if cross-modal and cross-platform data fusion can improve the predictive accuracy of personality. In the rest of this section, we describe in more detail the data sets used in the analysis, the methods for obtaining the features used in our results and the methodology for predicting personality traits.

### Data

**Fig 3:**
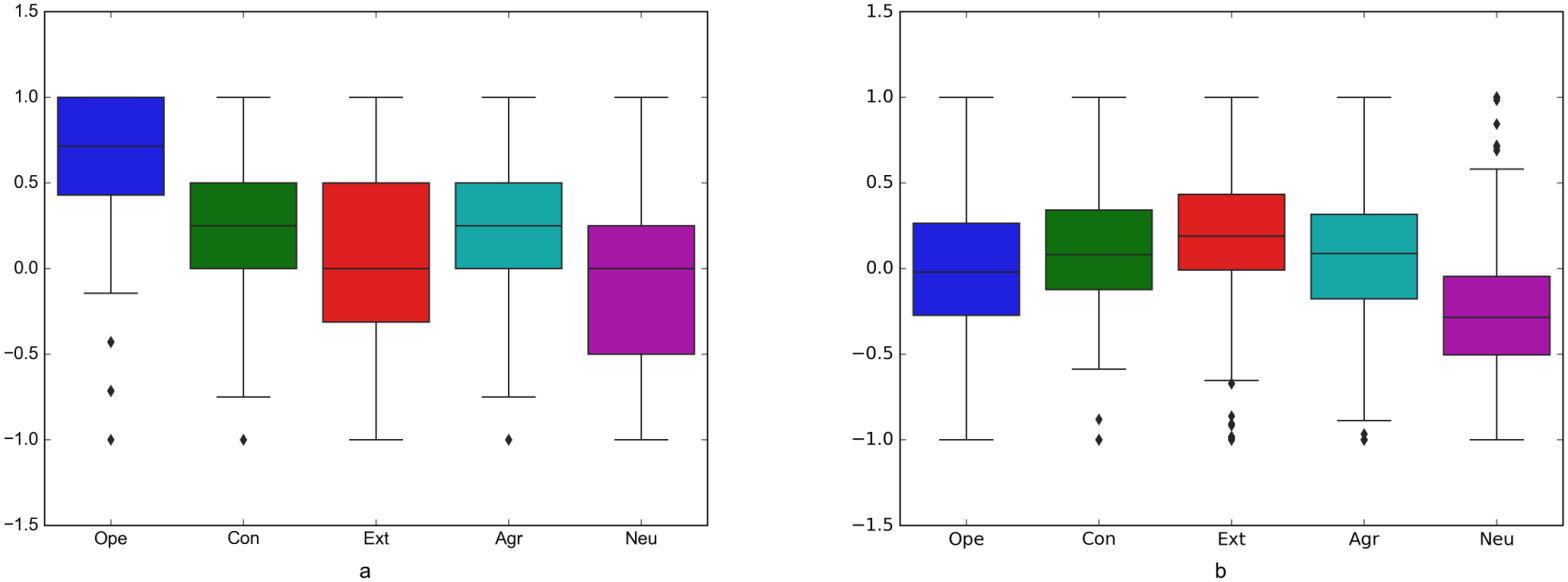
Distribution of different personality traits at the two data sets. (a) Psycho-Flickr and (b) Cross-Linked Flickr and Twitter.

**PsychoFlickr data set** This data set contains a set of self-assessed and perceived personalities for 300 random pro users from Flickr [47]. Pro users of Flickr are reportedly more likely to be passionate about photography [47]. In this paper, we use the Flickr API (https://www.flickr.com/services/api/) to extract profile images of those users and up to 300 of their posted and liked images. We collect in total 295 profile images, 72,997 posted and 60,001 liked images in this data set. Since we are interested in examining users’ personality, and not its perception, we use the self-assessed personality traits in this study. Table 2 shows the descriptive statistics and Fig 3 shows the distribution of different personality traits in this data set. We use this data set for the cross-modal analysis—to compare user personality prediction across different modalities (profile images, likes and posts).

**Table 2:** Descriptive statistics of the PsychoFlickr data set.

	*Flickr*		
Modality	Total #images	Average #images per user	Median #images per user
Posts	72,997	247	170
Likes	60,001	203	200
Profile Images	295	1	1

**Cross-linked Flickr-Twitter data set** This data set contains a set of 334 users having both Flickr and Twitter accounts and their estimated personality traits. All data was collected according to Twitter and Flikr’s terms of service and privacy conditions. We do not have personality computed via surveys for this data set, as these are very costly and time-consuming to administer. Hence, following previous work on personality analysis from profile images [33], we use an automatic text-regression method to assign each user scores for the Big Five personality traits [48]. The model was trained on a sample of over 70,000 users, using tokens and topics extracted from status updates as features, achieving a validation predictive performance of *r* ∼.35 on average for all five traits [48], which is considered a high correlation in psychology, especially when measuring internal states [49]. For each user, we downloaded up to 3200 of their most recent tweets using the Twitter API (https://dev.twitter.com/rest/public) to help with predicting personality traits. Table 3 shows the descriptive statistics of this data set. We use this data set for the cross-platform analysis.

**Table 3:** Descriptive statistics of the Cross-Linked Flickr and Twitter data set.

	***Flickr***		
**Modality**	**Total # images**	**Average # images per user**	**Median #images per user**
Posts	60,381	175	56
Likes	28,658	83	45
Profile Images	344	1	1
	***Twitter***		
**Modality**	**Total # images**	**Average # images per user**	**Median #images per user**
Posts	73,576	213	199
Likes	29,030	84	82
Profile Images	344	1	1

In building this data set, we selected the users who reported their Flickr profile in their Twitter description. Further, we use the Flickr API to extract profile, and up to 300 posted and liked images for each user, similar to the previous data set [47]. We collect a total of 334 profile images, 60,381 posted and 28,657 liked images on Flickr.

For the same set of users, we collected image data using the Twitter API—a total of 334 profile images, 73,576 posted and 61,197 liked images on Twitter. In order to obtain results comparable with the ones obtained on the Flickr data, we sub-sampled 29,030 liked images for the analysis. Fig 3 shows the distribution of different personality traits.

### Feature extraction

In order to study and interpret different modalities, we use two categories of features: colors and content. The former contains basic color information and the latter contains information extracted from the content of the image. For profile images, we use the features extracted from the profile image of the user and for liked and posted images we perform a mean feature pooling of all liked and posted images each across all images per user. The features used in this study are summarized in Table 4 and are described below.

**Table 4:** Description of features used in this work.

Feature Type	Dimension	Feature Name	Detailed Description
Color	1	Grayscale (binary)	if an image is grayscale or not. If the image is grayscale, then the rest of the features are not computed
	10	HSV statistics	Average and standard Deviation of hsv space, number of distinct hues, natural log of h_count
	12	Hue statistics	12 hue histogram (normalized, all 12 values sum up to 1)
	1	Pleasure (p)	Pleasure = 0.69∗Brightness+0.22∗Saturation
	1	Arousal (a)	Arousal = 0.31∗Brightness+0.60∗Saturation
	1	Dominance (d)	Dominance = 0.76∗Brightness+0.32∗Saturation
	6	6 Hue histogram	yellow, green, cyan, blue, magenta, red
Content features	1365	CNN object and scene probabilities	VGG_Net prediction on 1000 objects and 365 scene categories
	4096	CNN generic features	4096 dim penultimate layer features of VGG_Net
	1299	Imagga tags	list of Imagga tags for a set of images

**Color Features** Images are first converted to HSV space (Hue–Saturation–Value) as this provides a more intuitive representation of colors for users [50]. A pixel in the HSV space is characterized by three numbers: (1) *Hue*—the color type ranging between 0 and 360 degrees e.g., 0 is red, 60 is yellow, is green; (2) *Saturation*—the intensity of the color ranging from 0 to 1 e.g., 0 means no color; (3) *Value*—the brightness of the color ranging from 0 to 1 e.g., 0 represents black. Using the HSV representation, we first divide images into grayscale and color images. For color images, we calculate HSV statistics including mean and standard deviation of hue, saturation and value. We extract brightness and saturation as the mean of saturation and values respectively. An experimental study of colors established a linear relationship between saturation and brightness and the dimensional model of affect containing three factors: Pleasure, Arousal and Dominance [51, 52]. We also extract the hue histogram count for yellow, green, cyan, blue, magenta, red, the 12 color hue histogram, number of distinct hues (h_count) and its natural log (log_h_count). Out of the 32 dimensional vector we extract, some of these features have been applied in [47] to analyze personality of people who liked images on the PsychoFlickr data set (**Color**).

**Content Features** To represent image content, we used features from convolutional neural network trained on the Places data set [53] and tags derived using the convolutional neural network based Imagga automatic image tagging system.

Convolutional networks (ConvNets or CNNs) have recently enjoyed a great success in large-scale image recognition. A deep convolutional neural network architecture with 16-19 hidden layers named VGGnet is proposed in [53]. This classifier achieved the best results in the ImageNet Large Scale Visual Recognition Challenge 2014 in the object classification and localization challenge. We apply the proposed VGGnet model on our images. For a given image *x*_*i*_, the last fully connected layer of the VGGnet—called the penultimate layer—produces 4096-dimensional activations, which are the high-level features used to represent image *x*_*i*_ (**CNN_Gen**). In addition, we apply the model trained on 1000 object ImageNet tagset [54] and 365 standard scene categories [55] and use the prediction probabilities across all scene and object categories as features (**CNN_Obj**).

Images can have very diverse content beyond the ImageNet categories which have a limited taxonomy relative to the content of social media images (e.g. not including faces or common objects). We thus use the **Imagga** tagging API (http://docs.Imagga.com/#auto-tagging) as our content analysis engine, which was successfully used by past research [56]. We labeled all images with the Imagga Tagging API and generated for each image a *bag-of-tags* out of the top-10 predicted tags, based on the developers’ recommendations. We removed all tags that occurred less than 200 times in our largest data set, leaving us with 1,299 distinct tags.

### Analysis

In our experiments, we first provide an analysis that shows how accurate each different set of features is at the task of personality prediction. Then, we investigate which modality of interaction—profile, posted or liked images—is most predictive of users’ personality. Finally, we investigate which platform—Twitter or Flickr—is more predictive of users’ personality, and how cross-platform fusion impacts prediction performance.

In all our experiments we use linear regression with Elastic Net regularization [57] as our prediction algorithm. We tried *L*1 and *L*2 regularizers and Elastic Net regularization performed better as they combine both *L*1 and *L*2 norms. Results are reported on 10-fold cross validation measured by using Pearson correlation over the 10-folds. The same patterns hold when evaluating the results with Root Mean Squared Error for regression and we omit them for brevity. In all sections, feature combination is performed by training a linear ensemble over the individual prediction scores of each feature set. To test the significance of the models, the F-statistic (ANOVA) and the p-values are reported. All the experiments are done repeatedly with randomized dataset splits for 100 times and standard deviation for all of the results were found to be less than 0.001.

## Results

In this section we answer the research questions raised in the Introduction.

### Feature analysis

**Fig 4:**
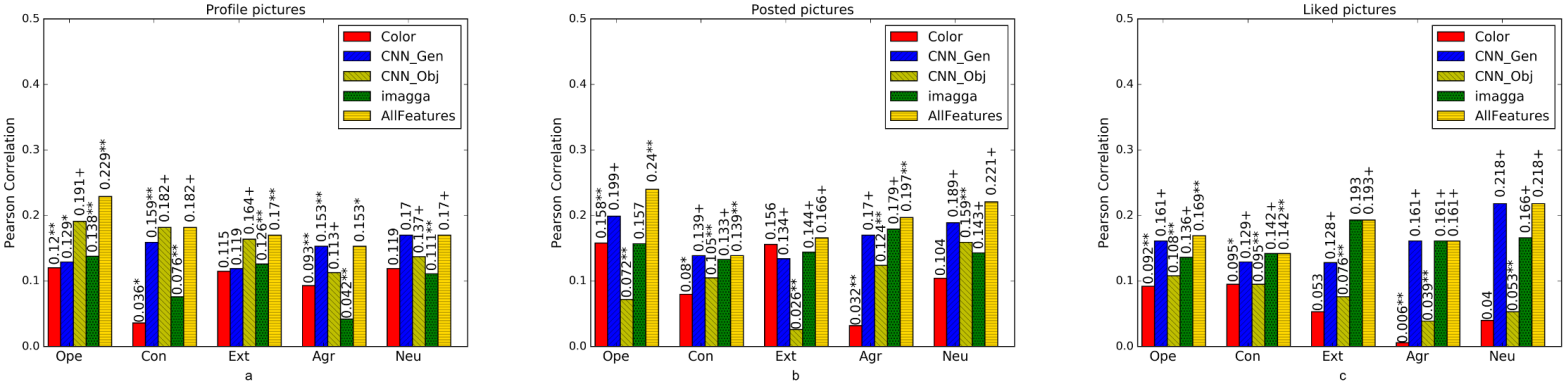
Prediction performance of models trained on different features: color, CNN generic features (CNN_Gen), CNN object and scene categories (CNN_Obj) and Imagga tags; extracted from (a) profile images, (b) posted images and (c) liked images measured in Pearson correlation on the PsychoFlickr data set. All Features denotes the performance of a model trained as linear ensemble of models trained on individual features. Significance of models is tested based on F-statistics (ANOVA); +: *p* < 0.05, *: *p* < 0.01, **: *p* < 0.001.

We compare the performance in predicting personality traits of the following sets of features, across each modality: colors, CNN_Gen features (from the penultimate layer of VGGnet proposed in [53]) and object/scene probabilities (from the final layer of the same network trained on [55]) and Imagga tags. In addition, we build a model that uses a combination of the features. Results are shown in Fig 4. The results show that:

(1) For profile images, CNN_Obj outperform other features for openness, conscientiousness, extraversion and neuroticism while CNN penultimate-layer features have the best performance only for agreeableness. In this modality, color features and Imagga tags have similar patterns. Their predictive performance is better for openness than conscientiousness and agreeableness. We observe that combining features leads generally to better results.

(2) For posted images, Imagga tags and CNN penultimate-layer features generally achieve the best predictive performance when compared to color and CCN categories. Imagga tags and CNN penultimate-layer features have nearly the same predictive performance for conscientiousness, extraversion and agreeableness and CNN penultimate-layer features slightly outperform others for openness and neuroticism. The overall better accuracy of CNN penultimate-layer features and Imagga tags is explainable by the fact that posted images contain a very diverse array of objects and subjects—as opposed to profile pictures—which are best captured by general image content features. CNN_Obj are not as good predictors probably due to the lack of diversity of the ImageNet categories, which do not include usual objects and subjects encountered in social media images.

(3) For liked images, Imagga and CNN penultimate-layer features achieve, similarly to posted images, the best predictive results on all personality traits. CNN penultimate-layer features outperform others in extraversion, neuroticism and openness, with the two achieving similar performance on the other three traits. Again, in this modality, color and CNN_Obj features follow similar patterns. Combining features does not add significantly to predictions on each trait, which demonstrates that all feature types capture similar information.

### Cross-modal analysis

**Fig 5:**
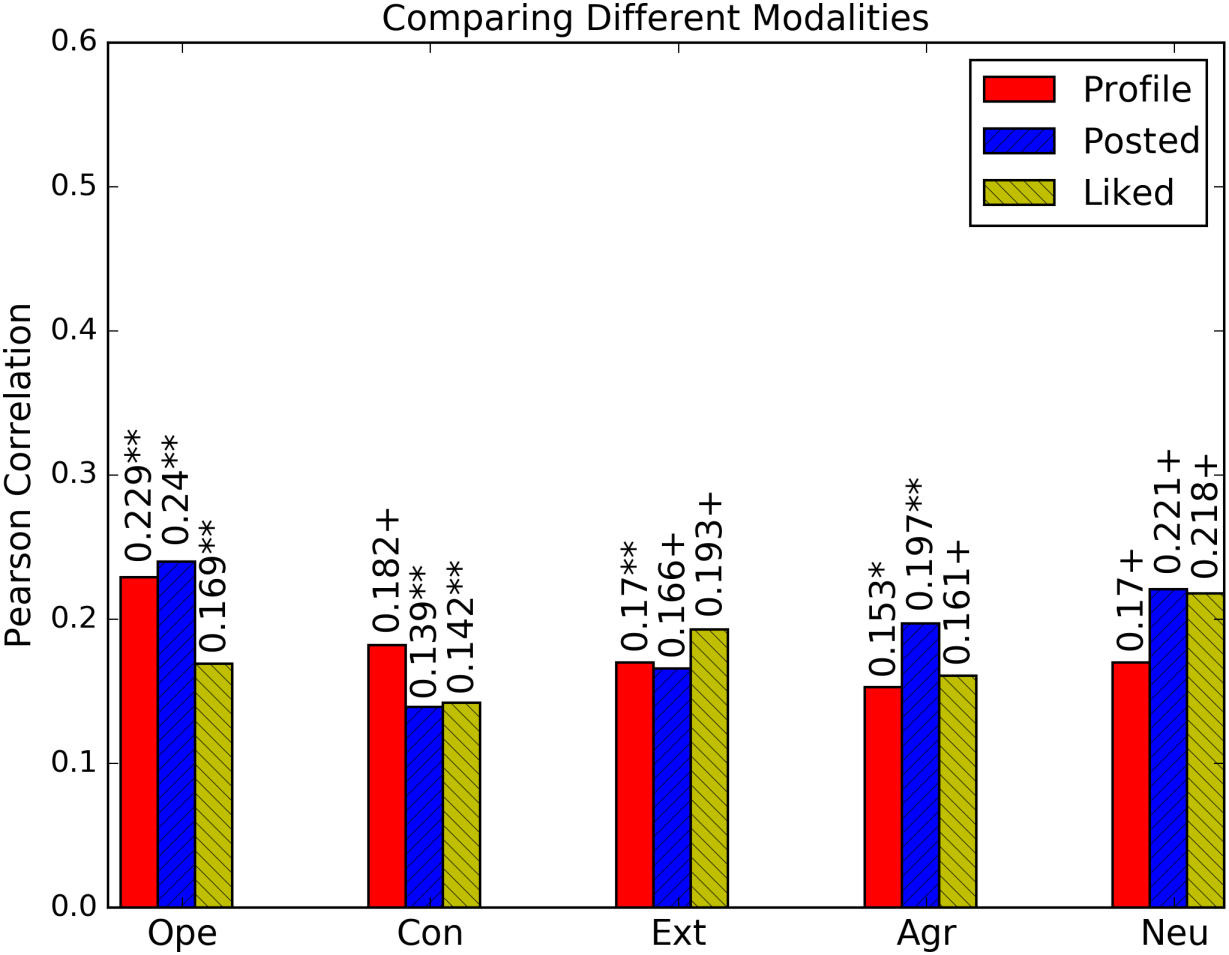
Prediction performance of models trained on features extracted from profile, posted and liked images based on Pearson correlation on the PsychoFlickr data set. Significance of models is tested based on F-statistics (ANOVA); +: *p* < 0.05, *: *p* < 0.01, **: *p* < 0.001.

In this experiment, we investigate the accuracy of models trained on different modalities at predicting personality traits. Models are trained using all features extracted from images. Results are presented in Fig 5 (showing a summarized view of results from Fig 4).

Profile pictures have the best performance in predicting conscientiousness and the lowest in predicting agreeableness. Posted images have overall the best predictive performance, being especially accurate at predicting agreeableness, openness to experience and neuroticism. This is not unexpected as posted images are more than a single profile image and represent the most direct way in which a user expresses his personality. Liked images do not achieve significantly better results than any of the other modalities on any personality trait, but are on par with posted images for neuroticism. Overall, this shows that liked images are not the most direct way of expressing personality, while profile images are surprisingly effective in personality prediction, taking into account that this only represents a single image.

### Cross-platform analysis

We investigate the predictive performance of images from two platforms in predicting different personality traits using the Cross-Linked data set where we have collected posted, liked and profile images from the same set of users on two different social media platforms: Twitter and Flickr.

**Fig 6:**
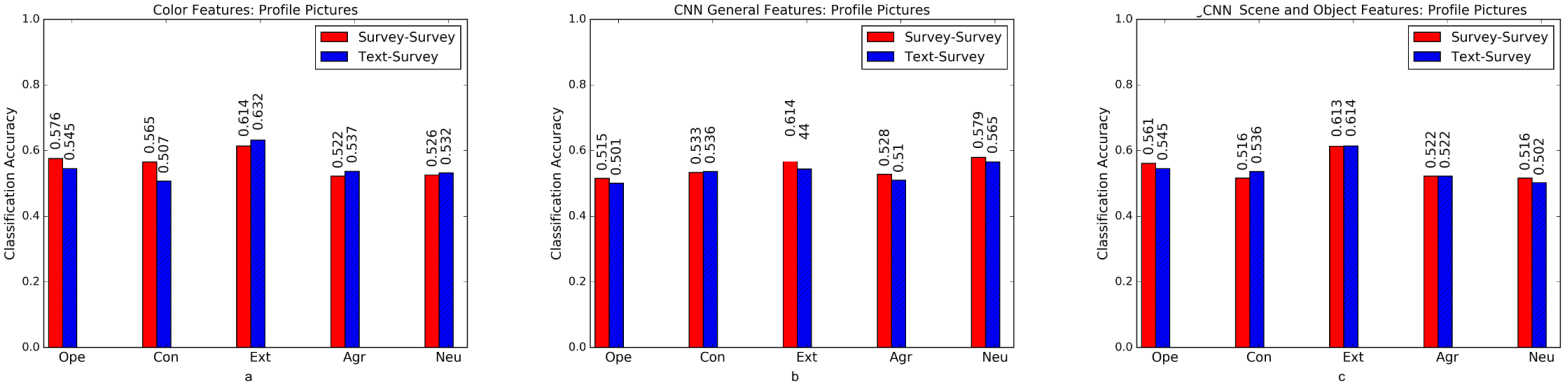
Profile images. Comparison of models trained on text-predicted labels (Crossed-Link Flickr and Twitter) and those trained on survey label data at predicting survey labels (Psycho-Flickr dataset) using (a) color features (b) CNN generic Features (c) CNN Probabilities on ImageNet Scene and Object Categories.

**Fig 7:**
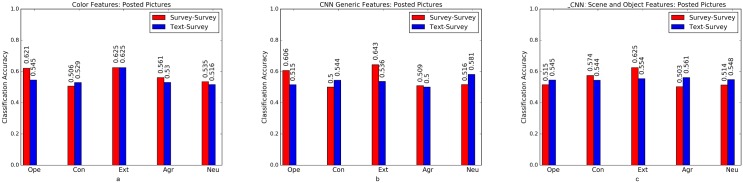
Posted images. Comparison of models trained on text-predicted labels (Crossed-Link Flickr and Twitter) and those trained on survey label data at predicting survey labels (Psycho-Flickr dataset) using (a) color features (b) CNN generic Features (c) CNN Probabilities on ImageNet Scene and Object Categories.

**Fig 8:**
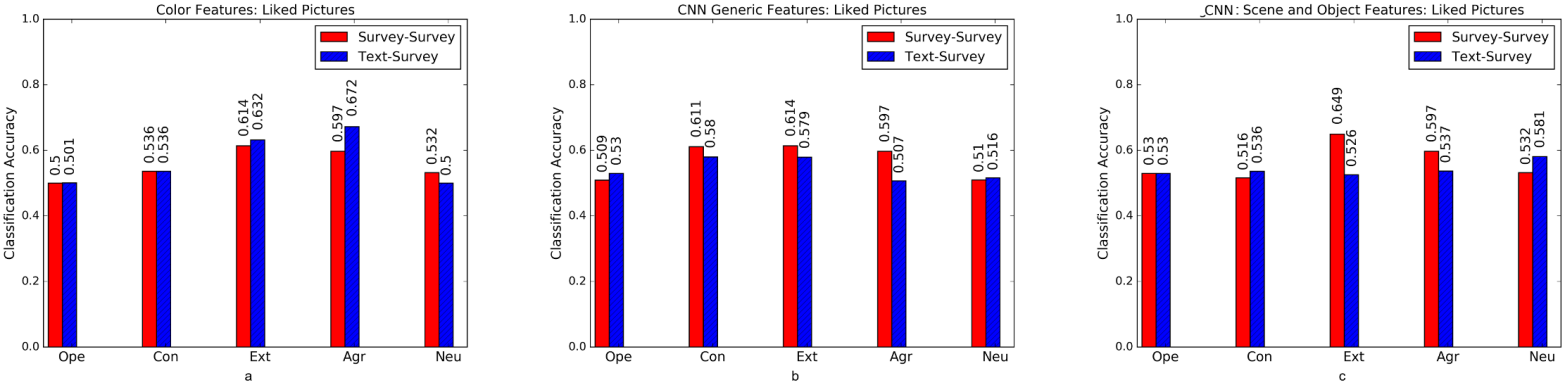
Liked images. Comparison of models trained on text-predicted labels (Crossed-Link Flickr and Twitter) and those trained on survey label data at predicting survey labels (Psycho-Flickr dataset) using (a) color features (b) CNN generic Features (c) CNN Probabilities on ImageNet Scene and Object Categories.

Psycho-Flickr consists of a set of users who answered the BFI survey [26] and Crossed-Linked Flickr and Twitter consists of a set of users with active accounts both on Flickr and Twitter. We used text mining approaches to predict personality traits for this set of users. To examine the robustness of text-predicted labels, we train models on Cross-Linked Flickr and Twitter data and test them on survey labeled personality traits of Psycho-Flickr dataset. We binarise the labels on both datasets using quartile split (as done by Segalin et al. [12]), divide the Cross-Linked Flickr and Twitter dataset into two splits (70% train and 30% test) for the analysis. Baseline accuracy here is 50%. We apply a combination of Random Forests and Support Vector Machine classifiers that have been used in computer vision and social science problems [58, 59]. The result are shown in Figs 6 to 8, where a comparison between models trained on text-predicted labels and tested on survey labels versus models trained and tested on questionnaire (survey) labels is presented. Models trained on text-predicted labels perform as well as models trained on survey labels, if not better in some cases, perhaps due to the large scale sample size used to train the text based model [28]. Further studies need to study this behavior using large-scale survey based samples.

**Fig 9:**
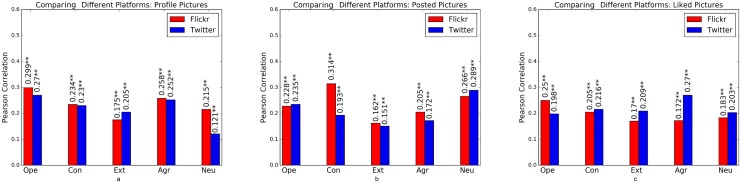
Prediction performance of different platforms for (a) profile, (b) posted, (c) liked images based on Pearson correlation on Cross-Linked Twitter and Flickr data set. Significance of models is tested based on F-statistics (ANOVA); +: *p* < 0.05, *: *p* < 0.01, **: *p* < 0.001.

Results using a combination of all feature types are shown in Fig 9 for each modality (i.e. profile pictures, posted and liked images) and platform, which we describe in more detail in the following paragraphs.

**Comparing Independent Modalities**: For profile images, the results are largely similar, with Flickr clearly outperforming Twitter only for neuroticism. For posted images, the performance is relatively similar for all traits except conscientiousness where Flickr data achieves better performance. For liked images, Twitter data is overall most predictive than Flickr, with the exception of openness to experience.

**Fig 10:**
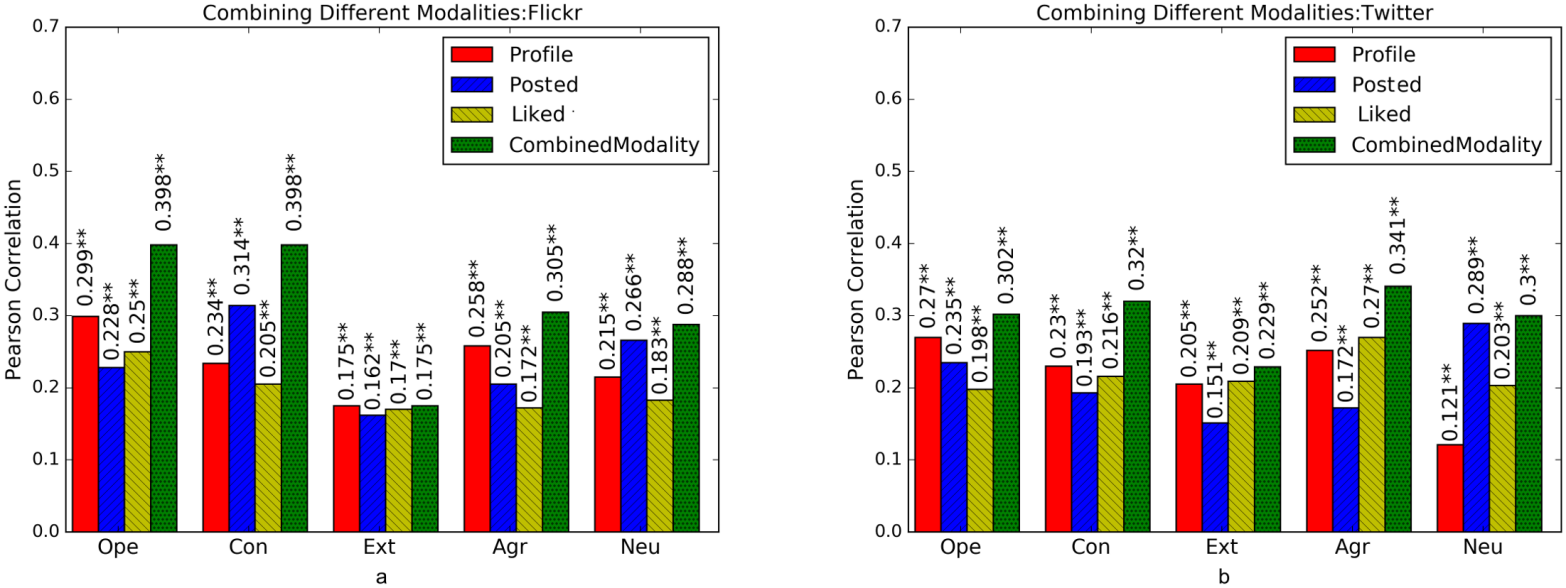
Prediction performance of combining different modalities (i.e. profile pictures, posted and liked images) versus using each modality separately on (a) Flickr and (b) Twitter based on Pearson correlation on Cross-Linked Twitter and Flickr data set. Combined Modality denotes the performance of a model trained as linear ensemble of models trained on individual modality. Significance of models is tested based on F-statistics (ANOVA); +: *p* < 0.05, *: *p* < 0.01, **: *p* < 0.001.

**Comparing Combined Modalities**: Next, we combine the three modalities (i.e. profile pictures, posted and liked images) to see if we can improve predictive results, thus exploring if these capture complimentary information and its extent. The results are shown in Fig 10. As seen in this figure, combining modalities always results in better predictive performance and in some cases, the improvement obtained is relatively large, for example in the case of openness to experience and conscientiousness and Flickr. For Twitter, the improvements are relatively smaller. This shows that in Flickr posting and liking images are more disparate actions, while on Twitter their content is more similar.

**Fig 11:**
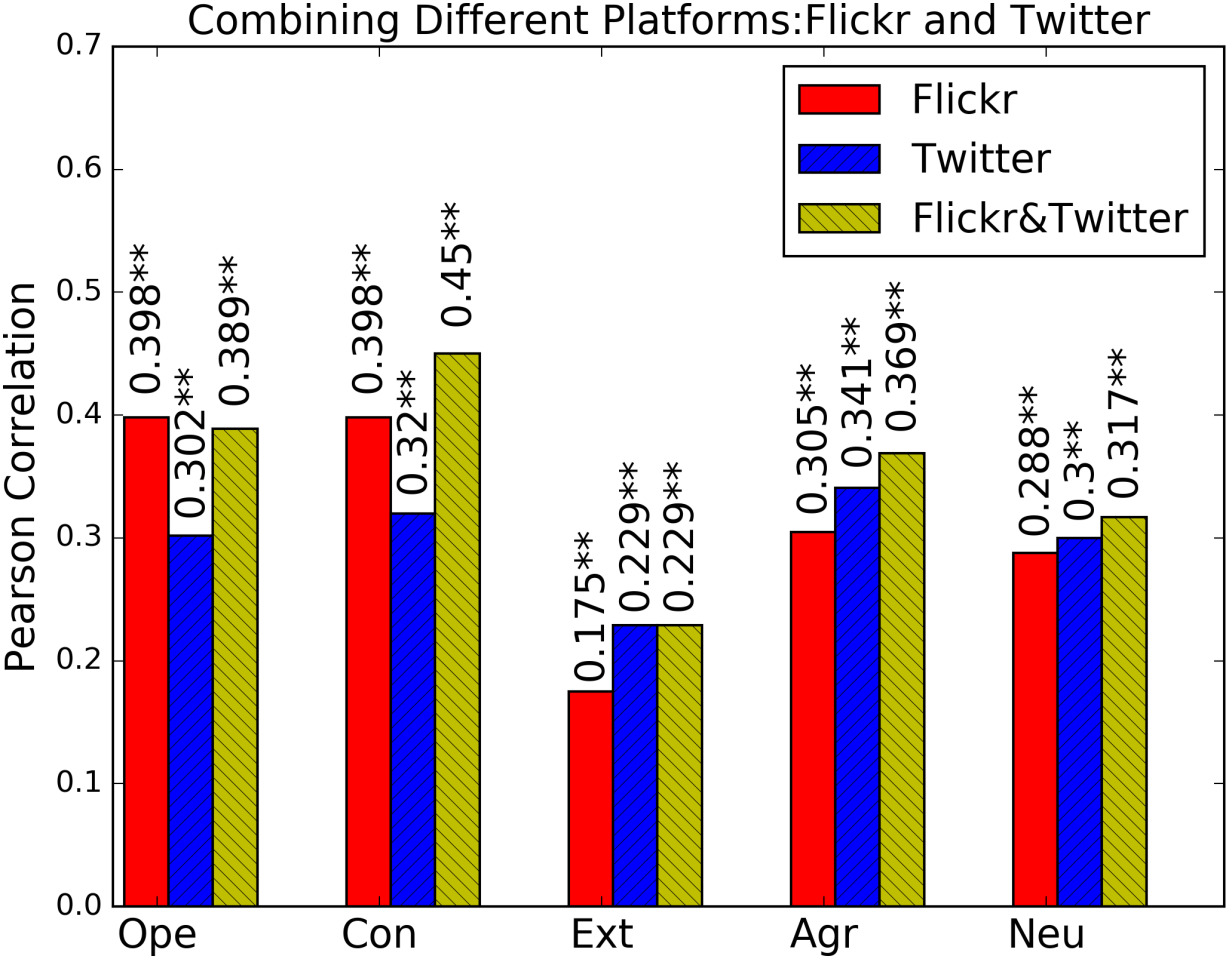
Prediction performance for combining different modalities and different platforms based on Pearson correlation on Cross-Linked Twitter and Flickr data set. Significance of models is tested based on F-statistics (ANOVA); +: *p* < 0.05, *: *p* < 0.01, **: *p* < 0.001.

**Comparing Combined Platforms**: Finally, we explore if combining information from both platforms can result in an additional boost in prediction performance. We achieve this by building a linear ensemble on top of the feature- and modality- ensembles build in the previous step. The results are shown in Fig 11. We can see that combining information from different platforms (Flickr and Twitter) can additionally slightly improve results, with the exception of extraversion. Overall, Flickr is more predictive of openness and conscientiousness, Twitter is more predictive in case of extraversion, and for agreeableness and neuroticism, the performance is similar. In absolute terms, conscientiousness is most predictive overall, followed by openness to experience and agreeableness. Extraversion is the least predictable personality trait.

## Discussion

The results of this work confirm the hypothesis that multiple interactions that users have with social media platforms such as choosing profile pictures, posting and liking images have predictive utility for automatic personality assessment of users, albeit with varying levels of performance; and combining different interaction types and platforms, although it involves more computation, can boost the prediction results. While posted images topped the performance in predicting personality followed by liked images and then profile pictures, profile pictures are a ubiquitous way for users to present themselves on social media, and they are usually considered public data which makes them easier to be accessed by automatic algorithms. Posted and liked images, on the other hand, are relatively more diverse in their content and automatic algorithms would need access to a larger set of such images across user’s posting timeline than liked pictures to make accurate predictions.

Posted images specially perform well in predicting openness to experience which stands for intellectual stimulation, willingness to explore new ideas, and similar traits. High prediction performance using posted images can be associated to prior research [60] that has shown two criteria for personality prediction to be successful—the environment in which users are must allow them to express the trait (termed as ‘Relevance’) and, the trait must be perceptible to others (termed as ‘Availability’), in this case automatic algorithms.

Liking images can be a consequence of multiple motivations including social factors or affective aspects such as reminding of positive events or ties with the people that have posted them. ‘Likes’ are a way users publicly and voluntarily express appreciation for content online [61]. As a result, users on several photo-sharing platforms create galleries of ‘favorite’ pictures which provides computer vision and social science researchers a strong source of data for analyzing users personality.

Difference in the online social networking platforms also is an important consideration for automatic methods to assess personality accurately. For example, Flickr is a social networking site that is used by people who do photography more professionally [47]. Instead, Twitter is a social media site on which users can share a diverse array of contents they are interested in [62]. Comparing both platforms showed that Twitter performance is noticeably higher in predicting agreeableness from liked images. The fact that agreeable people tend to evaluate content favorably is represented in twitter more than Flickr. We also find that Flickr has a higher performance at predicting conscientiousness from posted images, which corroborates the hypothesis that Flickr is used by people who do photography more professionally.

A lot of systems can benefit from personality detection. For example, dating websites can trying to match personalities of individuals before they meet each other [18]. Human Resources department could predict job satisfaction before hiring a potential employee. Recommender systems and commercial companies can improve their accuracy by recommending photos, movies or music, that have higher chance to make positive impressions on their users. Knowledge of a user’s personality also enables software developers to customize user interfaces [63].

This work provides multiple directions for future works. Psychological studies [64] show that biological and socio-demographic factors are parameters in shaping an individual’s personality; thus, adding socio-demographic features such as ethnicity, language, cultural and financial background, family size can potentially provide more insight. Further, it would be interesting to study the information contained in social media usage which goes above and beyond socio-demographics. While in this work we could not delve into providing insight due to the restriction we had with the data size, future work on larger samples and developing more interpretable visual features can serve to both boost performance [38] and to provide more insight about the manifestation of personality online. Recently, methods based on Gaussian Processes have been recently used to improve personality [65] and demographic [66] prediction. Though they improve the state-of-the-art in user-trait modeling, they are unlikely to significantly impact the answers to the research questions stated in our paper. We will leave improving the prediction accuracy for future work.

The feasibility of social-media-based assessment of personality traits also raises ethical questions. Organizations with vested interests could exploit this information, for example, to potentially influence people towards their agenda using social media. Data protection and ownership frameworks are needed to make sure the data is not used against the users’ interest. Few users realize the amount of psycho demographic information that can be gleaned from their digital traces, so transparency about the derived indicators should be part of ethical and policy discourse [67].

## Conclusion

We carried out a cross-modal and cross-platform study using images posted on social media. We used a wide range of color and semantic features extracted from images to analyze how different features can be applied to predict Big Five personality traits.

Posted images are generally more predictive than liked images and profile images, albeit profile images obtained good results given that this only represents a single image. Overall, semantic features from CNN and Imagga are the best feature types for modelling the content of posted and liked images.

Results on our novel cross-linked data set showed that Flickr provides overall better signal than Twitter for predicting personality traits. Combining modalities is shown to generally improve predictive performance especially in the case of Flickr, showing that the multiple modalities encode more complimentary information than they do on Twitter. Finally, combining Flickr and Twitter information largely improves results, albeit not with wide margins. Overall, our analysis shows that conscientiousness and openness to experience are the most predictable personality traits from images posted online.
